# Microphysiological Modeling of the Structure and Function of Neuromuscular Transmitter Release Sites

**DOI:** 10.3389/fnsyn.2022.917285

**Published:** 2022-06-13

**Authors:** Rozita Laghaei, Stephen D. Meriney

**Affiliations:** ^1^Biomedical Applications Group, Pittsburgh Supercomputing Center, Carnegie Mellon University, Pittsburgh, PA, United States; ^2^Department of Neuroscience, Center for Neuroscience, University of Pittsburgh, Pittsburgh, PA, United States

**Keywords:** computational modeling, motor nerve terminal, active zone, neuromuscular junction, MCell

## Abstract

The general mechanism of calcium-triggered chemical transmitter release from neuronal synapses has been intensely studied, is well-known, and highly conserved between species and synapses across the nervous system. However, the structural and functional details within each transmitter release site (or active zone) are difficult to study in living tissue using current experimental approaches owing to the small spatial compartment within the synapse where exocytosis occurs with a very rapid time course. Therefore, computer simulations offer the opportunity to explore these microphysiological environments of the synapse at nanometer spatial scales and on a sub-microsecond timescale. Because biological reactions and physiological processes at synapses occur under conditions where stochastic behavior is dominant, simulation approaches must be driven by such stochastic processes. MCell provides a powerful simulation approach that employs particle-based stochastic simulation tools to study presynaptic processes in realistic and complex (3D) geometries using optimized Monte Carlo algorithms to track finite numbers of molecules as they diffuse and interact in a complex cellular space with other molecules in solution and on surfaces (representing membranes, channels and binding sites). In this review we discuss MCell-based spatially realistic models of the mammalian and frog neuromuscular active zones that were developed to study presynaptic mechanisms that control transmitter release. In particular, these models focus on the role of presynaptic voltage-gated calcium channels, calcium sensors that control the probability of synaptic vesicle fusion, and the effects of action potential waveform shape on presynaptic calcium entry. With the development of these models, they can now be used in the future to predict disease-induced changes to the active zone, and the effects of candidate therapeutic approaches.

## Introduction

### The NMJ as a Model Synaptic System

Synaptic communication at neuromuscular junctions (NMJs) occurs at specialized sites called active zones (AZs). The NMJ AZ structure and function represents an ideal model system that has been studied using a wide variety of experimental techniques including electrophysiology, freeze-fracture electron microscopy, electron microscopic tomography, and immunohistochemistry. Not only is the NMJ an easily accessible synapse for these experimental studies, but the well-organized structure and organization of AZs at neuromuscular synapses facilitates comparisons of AZ structure and function. These studies have helped to shape our understanding of how the structural and functional organization of presynaptic AZs are coupled. Before discussing specific use of MCell computer modeling at this synapse, we will first describe the experimental work that constrains MCell models of the NMJ AZ, and the gaps in our understanding for which MCell modeling can be applied.

### The Control of Transmitter Release at NMJ AZs

**Figure 1:**
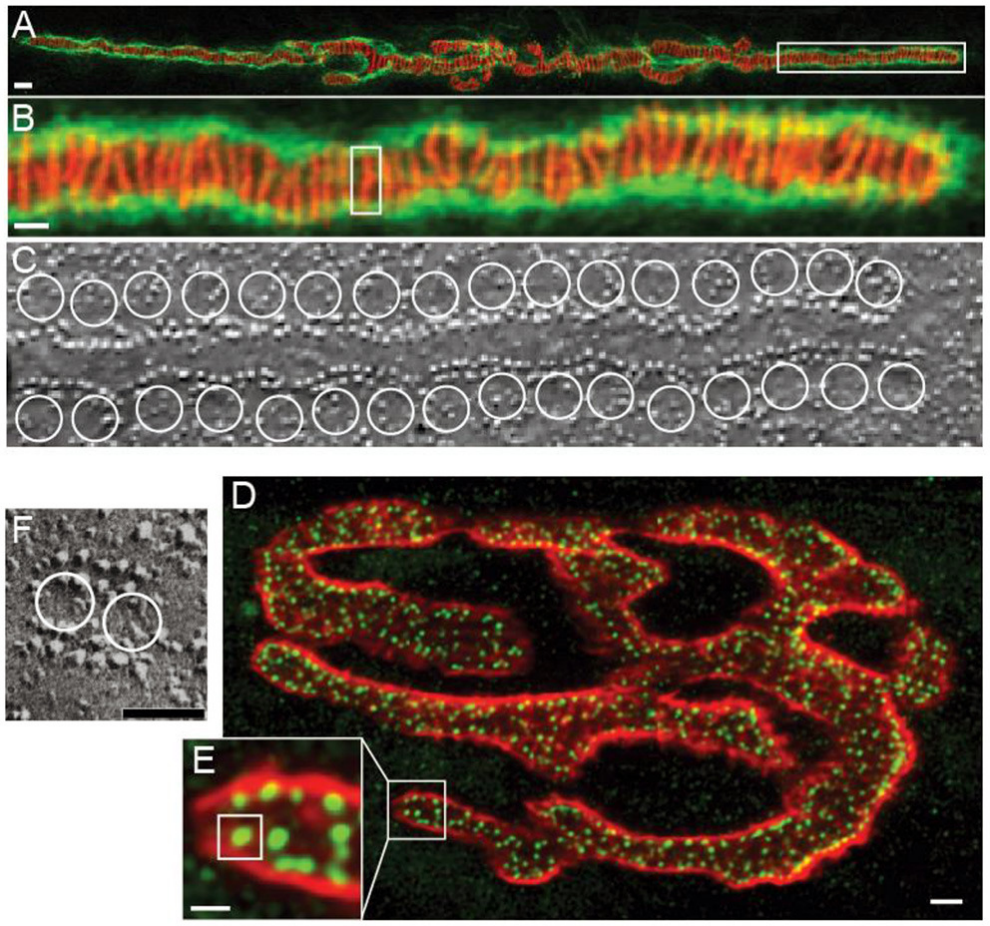
Active zones at frog and mouse NMJs. **(A)** Confocal image of a single frog NMJ stained with Alexa 594 a-bungarotoxin (red) and peanut agglutinin (green). The a-bungarotoxin staining predicts the location of AZs because this toxin binds to the rows of acetylcholine receptors that are immediately adjacent to the presynaptic active zones. **(B)** Enlargement of the portion of the image in A that is contained in the white box to show the parallel arrangement of long linear AZs. A single AZ is shown in the white box. **(C)** Freeze-fracture electron micrograph of a single AZ showing the characteristic two double rows of intramembranous particles that are thought to represent AZ proteins. White circles are drawn to represent the predicted position of docked synaptic vesicles. **(D)** Confocal image of a single mouse NMJ stained with Alexa 594 a-bungarotoxin (red) and an antibody to the protein bassoon (green). The bassoon staining identifies the location of AZs. **(E)** Enlargement of the portion of the image in **(A)** that is contained in the white box to show the small punctate AZs. A single AZ is shown in the white box. **(F)** Freeze-fracture electron micrograph of a single AZ showing the characteristic two double rows of intramembranous particles that are thought to represent AZ proteins. White circles are drawn to represent the predicted position of two docked synaptic vesicles. Adapted from Heuser et al. (1979); Fukuoka et al. (1987), and Laghaei et al. (2018).

The frog NMJ has been used for decades as a model synapse not only for specific features of neuromuscular synaptic transmission, but also for the study of conserved mechanisms of neurotransmission in general (Del Castillo and Katz, 1953, 1954a,b, 1956; Katz and Miledi, 1965a,b; Tarr et al., 2013). A single frog NMJ contains ~600 AZs (Figure 1A) (Laghaei et al., 2018). Each AZ is a highly organized structure consisting of two long rows of 20–40 docked synaptic vesicles. The gap between these rows of synaptic vesicles contains two highly ordered double rows of 200–250 intramembranous particles (Figure 1C) (Heuser et al., 1974, 1979; Pawson et al., 1998). Despite the presence of 12,000–24,000 docked vesicles in the ~600 AZs, a presynaptic action potential triggers the release of neurotransmitter from only about 200–500 synaptic vesicles (mean = 415) (Laghaei et al., 2018). This means that during an action potential, each AZ has a 0.67 probability of a single synaptic vesicle fusion event. Assuming an average of 30 docked synaptic vesicles per AZ, the probability that any given synaptic vesicle will fuse and release neurotransmitter during an action potential is only 0.022. However, since there are thousands of docked synaptic vesicles, this low fusion probability per vesicle still results in the release of more total transmitter following each presynaptic action potential than is required to elicit a muscle contraction; this is often called the “safety factor” (Wood and Slater, 2001).

The mouse NMJ is also often used as a model synapse, as it is considered a useful animal model for human neuromuscular transmission. Whereas, the frog NMJ has long finger-like nerve terminals with long linear AZs, the mouse NMJ has a “pretzel” shape with much smaller short linear AZs (Figure 1D). Each AZ contains a row of about two docked synaptic vesicles surrounded on both sides by double rows of intramembranous particles, containing a total of ~20 particles (Figure 1F) (Fukuoka et al., 1987; Nagwaney et al., 2009). The average mouse NMJ contains ~700 of these smaller AZs (Ruiz et al., 2011; Chen et al., 2012; Laghaei et al., 2018), each placed ~500 nm apart (Ruiz et al., 2011). During a single presynaptic action potential, the mouse NMJ releases ~160 vesicles of neurotransmitter. This corresponds to a 0.22 probability of release from any given AZ during an action potential, and a 0.11 probability of release per synaptic vesicle (Laghaei et al., 2018). However, since there are about 1,400 docked synaptic vesicles, this low fusion probability per vesicle still results in the release of more transmitter following each presynaptic action potential than is required to elicit a muscle contraction (the safety factor) (Wood and Slater, 2001).

### Calcium Dynamics That Regulate AZ Function

Calcium imaging has provided valuable insight into how calcium ions trigger vesicle fusion at vertebrate NMJs (Rizzoli and Betz, 2002; Wachman et al., 2004; Shahrezaei et al., 2006; Luo et al., 2011, 2015; Samigullin et al., 2014, 2017; Nurullin et al., 2019). The results of these studies suggest that of the 200–250 intramembranous particles in the frog AZ, only 30–40 of them are predicted to be voltage-gated calcium channels (VGCCs), which is similar to the number of docked synaptic vesicles at these AZs. This suggests a one-to-one relationship between docked synaptic vesicles and VGCCs in the frog AZ. Furthermore, calcium imaging resulted in the conclusion that VGCCs in the frog AZ have only a 0.2 probability of opening during an action potential (Luo et al., 2011, 2015). Thus, only ~7 VGCCs would be expected to open in each AZ of the frog NMJ during a presynaptic action potential. This paucity of VGCCs combined with the low probability that VGCCs opening during an action potential provides one mechanism which can explain the low probability of fusion for synaptic vesicles (Shahrezaei et al., 2006; Luo et al., 2015). A second low probability step in calcium triggered vesicle fusion at the frog NMJ is the probability that calcium ion flux through an open channel will trigger a nearby docked synaptic vesicle to fuse. This probability has been estimated to be about 5% (Luo et al., 2015). Taken together, these two low probability events: VGCC opening in an AZ, and calcium-triggered vesicle fusion by the calcium ion flux through an open channel, are thought to underlie the resulting very low probability of vesicle release per AZ. The entire NMJ is known to be strong and reliable because it is constructed of hundreds of such low probability release sites where only a small subset of these are needed to release sufficient transmitter to bring the post-synaptic muscle cell to threshold. This sparse usage of AZs during any one action potential is thought to contribute to the reliability of the synapse as a whole by ensuring that, even during normal bouts of repeated stimulation, the synapse will not become depleted of docked synaptic vesicles.

Despite the wealth of information that calcium imaging has provided, due to the characteristics of the dyes used to image the dynamics of calcium ions, imaged signals are dominated by the relatively slow kinetics of the dye. As such, the actual kinetics of calcium ions diffusing within the nerve terminal cytoplasm cannot be directly measured. Based on experimental work and prior modeling efforts, it is hypothesized that after the opening of individual calcium channels, the diffusing calcium ions form small local gradients initiating near the mouth of open channels where calcium concentration is very high locally but falls off steeply with distance away from the channel due to diffusion and cytoplasmic calcium buffering. Consequently, the distance between presynaptic calcium channels and docked synaptic vesicles, and the relative arrangement and density of VGCCs and synaptic vesicles in the AZ are key determinants of the signaling properties of synapses and can play significant roles in spatiotemporal calcium dynamics and plasticity of neurotransmitter release (Neher, 1998a,b; Meinrenken et al., 2002; Eggermann et al., 2011). The hypotheses described above for how VGCCs function and how calcium ions trigger synaptic vesicle fusion can be tested using MCell models of these synapses where each calcium ion in the model can be tracked directly (described below).

### Ultrastructure of NMJ AZs: Freeze Fracture and EM Tomography

The classic experiments that defined the organization of AZ transmembrane proteins at the frog NMJ involved a rapid freeze-fracture approach (Heuser et al., 1974, 1979; Heuser and Reese, 1981). These early experiments rapidly froze the frog NMJ at different times relative to stimulating the motor nerve to not only characterize the resting AZ structure, but also to document changes to that structure during the process of synaptic vesicle exocytosis leading to transmitter release. Freeze-fracture replicas of these tissues defined the fine structure of the frog AZ which was characterized by two double rows of intramembranous particles in a highly ordered array (Figure 1). Subsequently, it was proposed that at least some of these intramembranous particles represented the location of presynaptic AZ VGCCs (Pumplin et al., 1981; Farinas et al., 1993). Based on pharmacological effects on transmitter release, calcium imaging, and toxin labeling, it was also proposed that some of the other intramembranous particles within the AZ were calcium-activated potassium channels (Robitaille et al., 1993). Subsequently, electron microscope tomography has been used to obtain a three-dimensional representation of the AZ fine structure in both frog and mouse NMJs (Harlow et al., 2001, 2013; Nagwaney et al., 2009; Szule et al., 2012; Jung et al., 2016, 2018). For these experiments, a fixed NMJ AZ was imaged in a transmission electron microscope from many different angles to allow the construction of a three-dimensional image. This resulted in a high resolution (2–20 nm) image that was used to determine the three-dimensional ultrastructure of sub-cellular compartments within the AZ (Ress et al., 2004). These ultrastructural studies were important for the development of MCell models of the NMJ AZ as they provide constraints on the placement of model components (described below).

### Calcium Sensors on Synaptic Vesicles

Experimental information on the calcium sensor proteins that translate elevated intraterminal calcium ions into synaptic vesicle fusion has also provided important constraints for modeling calcium-triggered vesicle fusion at the NMJ. Because calcium-triggered secretion is believed to be highly conserved between species and synapses, experimental work in many model systems has been used to inform the development of models of calcium sensor proteins. Based on prior knock-out experiments, synaptotagmin-1 (syt-1) has been implicated as the primary sensor for fast, synchronous calcium-triggered vesicle fusion (Geppert et al., 1994; Chapman, 2002; Nishiki and Augustine, 2004; Maximov and Sudhof, 2005). A second synaptotagmin family member, synaptotagmin-7 (syt-7), is also thought to be important in the presynaptic nerve terminal (Jackman et al., 2016; Turecek et al., 2017). Calcium binding to synaptotagmins occurs at two cytoplasmic C2 domains called C2A and C2B. The C2 domains in syt-1 bind calcium ions faster and with lower affinity than the C2 domains in syt-7 which bind calcium more slowly and at higher affinity (Jackman et al., 2016; Turecek et al., 2017). Following a single action potential, or during very low frequency action potential activity, the brief microdomain (localized volume of high intracellular calcium ions near the mouth of several open VGCCs) effectively activates syt-1. During high frequency action potential activity, syt-7 primarily contributes to asynchronous vesicle fusion and short-term plasticity (Bacaj et al., 2013; Jackman et al., 2016; Turecek et al., 2017). This is because syt-7 has a calcium binding off rate of ~60 times slower than that of syt-1 (Jackman and Regehr, 2017; Turecek et al., 2017). Both calcium sensors, syt-1 and syt-7, are thought to be present in the AZ, with syt-1 known to be located on synaptic vesicles, and syt-7 hypothesized to be a plasma membrane protein (Sudhof, 2012; Bacaj et al., 2013; Radhakrishnan et al., 2021). Taken together, the experimental results presented above have provided important constraints on the development of computational models of NMJ AZs. Because the details of how calcium ions diffuse within the nerve terminal cannot be imaged accurately when reporter dye kinetics dominate the measurements, and because the specific binding of calcium ions to calcium sensors on synaptic vesicles cannot be accurately measured experimentally at intact synapses, it is still debated exactly how these synaptotagmins control the process of synaptic vesicle fusion (Meriney et al., 2014). One particular unknown is the number of synaptotagmin proteins of both subtypes (syt-1 and syt-7) that have to bind calcium to effectively trigger synaptic vesicle fusion under different conditions. Therefore, the development of hypotheses as to how syt-1 and syt-7 trigger vesicle fusion at the NMJ can be aided by realistic diffusion-reaction simulations using MCell.

### Calcium Buffering in Motor Nerve Terminals

As mentioned in the previous sections, spatiotemporal calcium ion dynamics after VGCC opening play an important role in controlling the probability of synaptic vesicle fusion. The presence of calcium ion buffers within the nerve terminal impacts the concentration of free calcium ions and the buffered diffusion of these ions away from sites of entry since it is thought that 95% of calcium ions become bound within 10–50 nm of their site of entry into the nerve terminal (Neher, 1995). The endogenous buffers within nerve terminals that bind calcium can include mobile buffers (i.e., calbindin, calretinin, parvalbumin, and calmodulin) as well as fixed buffers associated with organelles (mitochondria and endoplasmic reticulum) or cytoskeletal-associated proteins. Fixed calcium ion buffers tend to reduce diffusion, while mobile buffers can enhance the spread of calcium ions. However, the properties, concentration, and identity of calcium buffers are not known for many presynaptic terminals. Using a combination of experimental and modeling approaches, the buffer concentration has been estimated at several synapses to be somewhere between 200 uM and 20 mM (Tank et al., 1995; Gabso et al., 1997; Sinha et al., 1997; Sinha and Saggau, 1999; Burnashev and Rozov, 2005; Schneggenburger and Neher, 2005). At the frog NMJ, the mobile buffer has been estimated at 200 uM and the fixed buffer at between 7 and 10 mM (Suzuki et al., 2000; Bennett et al., 2007; Samigullin et al., 2014, 2017). These studies provide a framework for constraining buffer parameters in MCell models.

### Short-Term Synaptic Plasticity

With repeated motor nerve stimulation at frequencies above 1 Hz, there are changes in the magnitude of transmitter released due to the combined effects of residual intraterminal calcium left behind by the preceding stimulation, and docked synaptic vesicle depletion that can accumulate during high frequency stimulation (Zucker and Regehr, 2002). The effect of residual calcium ions is to potentiate subsequent transmitter release, and the effect of vesicle depletion is to depress subsequent transmitter release. The balance of these two influences determines the final effect on synaptic transmission. Because MCell models of the NMJ track each calcium ion that enters the nerve terminal model space as they interact with the proteins present, and each synaptic vesicle fusion event, this modeling platform is ideal to study short-term synaptic plasticity mechanisms.

## Microphysiological Modeling at the NMJ AZ

### The MCell Simulation Tool

The MCell (www.mcell.org) simulation tool is a Monte Carlo reaction-diffusion modeling tool that uses spatially realistic 3-dimensional geometries and allows realistic synaptic microphysiology simulations. It specifically tracks each molecule in the system in space and time as they diffuse and interact with other molecules (Stiles et al., 1999; Kerr et al., 2008). To build 3-dimensional geometries to be used in MCell simulations, a graphical interface (Cell Blender) can be used. MCell and CellBlender are both open-source software packages. Since MCell simulations are stochastic, for each data point, 5,000–10,000 MCell simulations are usually performed to ensure reliable statistical averages. To accurately represent diffusion and reactions within the simulation, the time step for all simulations is 10 ns. During each run, action potential-driven rate constants cause VGCCs to open according to a channel gating scheme (Dittrich et al., 2013). When VGCCs open in the simulation as driven by this gating scheme, calcium ions are generated at the mouth of these open channels at a rate based on the conductance of the channel, and then these ions diffuse into the 3-dimensional model space where they can bind to, and unbind from, calcium buffer molecules and calcium sensors sites (modeled reaction sites for syt-1 and syt-7) located on synaptic vesicles. MCell tracks all calcium ions in the presynaptic space and keeps track of the origin of all calcium ions that contributed to the fusion of each synaptic vesicle. Synaptic vesicle fusion in MCell models occurs based on a user defined fusion model, and when a predefined number and/or distribution of calcium ions are bound to vesicle sensor sites, the vesicle is considered by the simulation to be fused with plasma membrane. To obtain the fusion rate, the total number of fusion events were summed over the thousands of model seeds that were run, divided by the total number of vesicles in the model, and then divided by the number of action potentials delivered in the model seeds. To ease data management, MCell simulations are run with a compressed binary format (.bin.bz2) for output to enable efficient storage as the simulation output files can be very large (40–50 TB). The simulation results can then be analyzed using in-house scripts written in Go and Python.

### The Use of MCell to Probe NMJ AZ Structure and Function

A major advantage of MCell models of the NMJ AZ is that they can be heavily constrained by the wealth of prior experimental data described above. These data define the AZ structural organization (Heuser and Reese, 1981; Harlow et al., 2001; Nagwaney et al., 2009), action potential waveform (Ginebaugh et al., 2020; Ojala et al., 2021), VGCC function (DeStefino et al., 2010; Luo et al., 2011, 2015), presynaptic calcium buffer capacity (Samigullin et al., 2014), copy number and distribution of SNARE protein/synaptotagmin complexes at each docked synaptic vesicle (Chapman, 2002; Han et al., 2004; Radhakrishnan et al., 2021), binding kinetics for synaptotagmin calcium sensors that trigger transmitter release (Turecek et al., 2017), the probability of vesicle fusion following an action potential (Luo et al., 2015), and short-term synaptic plasticity of transmitter release (Tarr et al., 2014; Laghaei et al., 2018). As a result, there are very few free parameters in these models which permits a constrained computational approach to outstanding issues in AZ function, and an examination of the impact of structural iterations intended to explore the parameter space for key issues in AZ structure and function.

### Calcium Sensors Responsible for Triggering Transmitter Release and Short-Term Synaptic Plasticity

**Figure 2:**
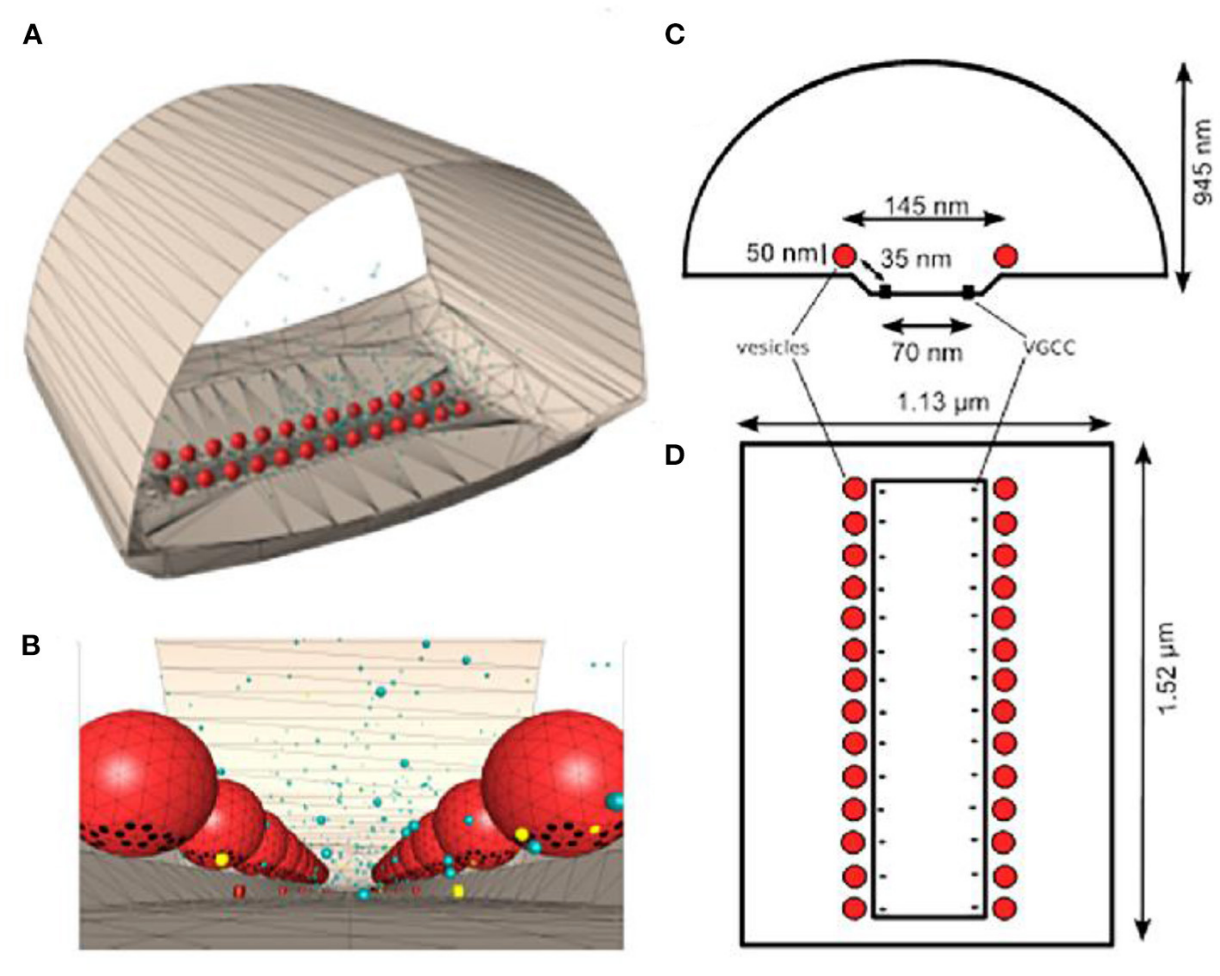
Frog MCell model. **(A)** MCell model of one AZ from the frog NMJ (equivalent to one linear red stained AZ in Figure 1B. **(B)** Enlarged view from within the frog MCell AZ model near the base of docked synaptic vesicles (red spheres) that includes synaptotagmin-1 binding sites (black dots). Calcium ions are shown as free (yellow dots) or bound to calcium buffer (blue dots). The 3-D models in **(A,B)** were created *via* CellBlender based on ultrastructural data from the adult frog NMJ. **(C)** Cross-section diagram of the distribution of VGCCs (black dots) and docked synaptic vesicles (red circles). **(D)** top view diagram of 26 N-type VGCCs; black dots) positioned in a 1:1 stoichiometric relationship with docked synaptic vesicles (red circles). Adapted from Dittrich et al. (2013).

**Figure 3:**
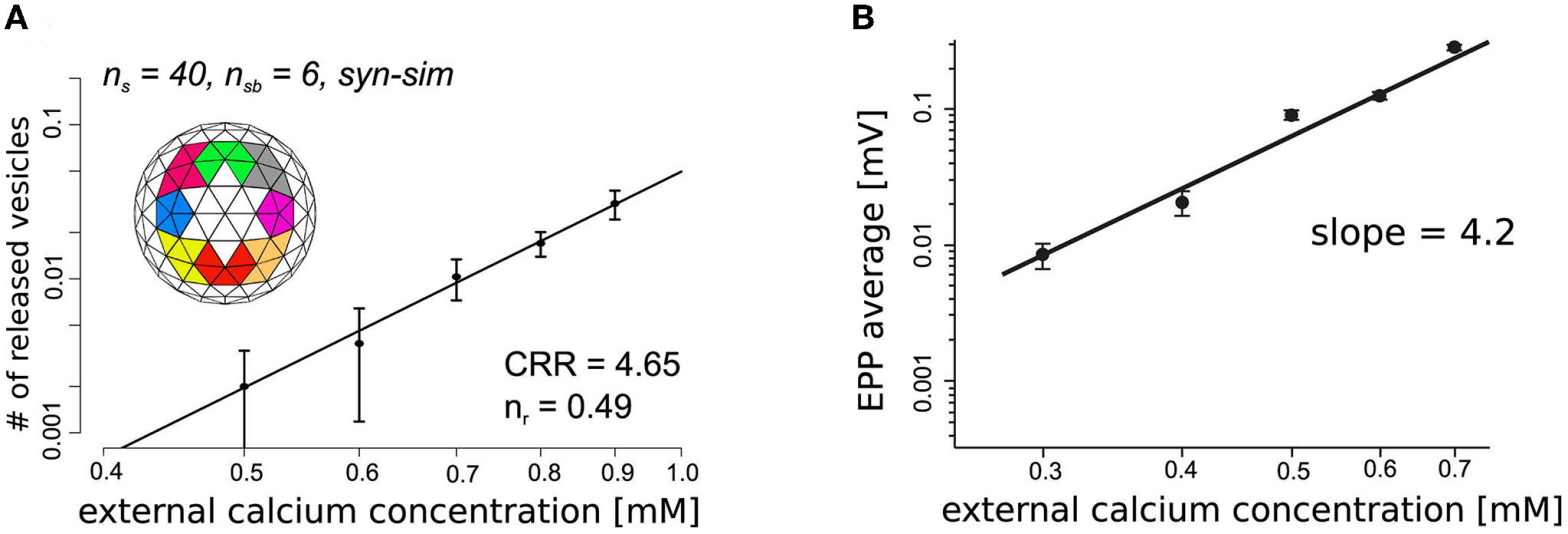
Prediction of fourth order calcium-release relationship using excess calcium ion binding sites with no inherent cooperativity. **(A)** Plot of predicted vesicle release probability at varying extracellular calcium concentrations using an MCell model with 40 calcium ion binding sites (n_s_) per vesicle (inset) but requiring only 6 of these binding sites to be simultaneously occupied (n_sb_) to trigger vesicle fusion. The model data display a roughly fourth order relationship (CRR = 4.65) when plotted on a log scale. Under these model conditions, the probability of release per AZ (n_r_) was 0.49, closely approximating the experimentally determined value (0.67). **(B)** Plot of experimental endplate potential amplitude data recorded using microelectrode techniques from a frog nerve-muscle preparation as the concentration of extracellular calcium was varied. When plotted on a log scale, these data display a fourth order relationship (slope = 4.2). Adapted from Dittrich et al. (2013).

One of the key unknowns at all synapses is the total number of calcium sensors associated with SNARE proteins at each docked synaptic vesicle site, and the number of these sensors that are required to bind calcium ions to trigger transmitter release. Prior studies have provided evidence that there are between three and eight SNARE protein complexes around the base of each docked synaptic vesicle (Chapman, 2002; Han et al., 2004; Radhakrishnan et al., 2021), and assuming these are each associated with one syt-1 protein, MCell has been used to explore not only this range of syt-1 proteins positioned around the base of each synaptic vesicle, but also how many of these are required to bind calcium to trigger transmitter release (Dittrich et al., 2013). These studies led to the development of a model incorporating syt-1 sensors that could predict the magnitude of transmitter release (Figure 2). In addition, these early models also predicted the known fourth power relationship between extracellular calcium concentration and transmitter release (calcium-release relationship; CRR), and predicted that the CRR could emerge as function of an excess number of calcium binding sites in comparison with the number of calcium binding sites required to trigger fusion, without any inherent cooperativity in binding between these sites (Figure 3). This represented an intriguing alternative hypothesis to prior models that had assumed transmitter release was triggered by only four or five cooperative calcium ion binding sites (Bennett et al., 2000; Schneggenburger and Neher, 2000; Bollmann and Sakmann, 2005; Millar et al., 2005; Matveev et al., 2006; Shahrezaei et al., 2006; Pan and Zucker, 2009). This new hypothesis provided a mechanism by which the CRR could emerge based on an excess number of calcium ion binding sites, and was consistent with biochemical data demonstrating that vesicular syt-1 molecules contain two calcium ion binding domains (C2A and C2B) each of which have a total of five calcium binding sites (Ubach et al., 1998; Chapman, 2002), and that each vesicle may have up to 15 copies of syt-1 (Takamori et al., 2006; Mutch et al., 2011). Interestingly, although C2 domains in syt-1 may bind two or three calcium ions each, it is possible that only one calcium ion needs to bind to screen C2 domain charges such that lipid binding may occur (Sutton et al., 1995). In any event, MCell modeling is an ideal platform within which to explore these stoichiometric details and predict their impact on synaptic function. Using this approach, Dittrich et al. (2013) could predict synaptic function and the fourth order CRR using docked synaptic vesicles with 20–40 calcium ion binding sites (corresponding to four to eight syt-1 proteins) if only a subset of these binding sites (five or six) had to bind calcium ions to trigger synaptic vesicle fusion, with no *ad hoc* binding site cooperativity (Figure 3).

In order to predict short-term synaptic facilitation, studies using MCell models of the NMJ found that a second calcium sensor (beyond one based on the properties of syt-1) was required. Ma et al. (2015) predicted that short-term synaptic facilitation could be successfully modeled by a second calcium sensor protein with lower affinity and slower kinetics. Soon after this report, an experimental study demonstrated that syt-7 was the second calcium sensor that was required for synaptic facilitation at several types of synapses (Jackman et al., 2016). Interestingly, the MCell predicted on-rate and off-rate of the second sensor (Ma et al., 2015) was very consistent with the syt-7 rates reported later (Jackman et al., 2016; Turecek et al., 2017). Because of the particle-based nature of MCell simulations, Ma et al. (2015) were able to evaluate the fractional contribution of each VGCC in the NMJ AZ to the release of each docked synaptic vesicle. For one action potential, on average only two channels contributed to each vesicle fusion event, in agreement with previously reported values (Shahrezaei et al., 2006). During repeated action potentials at high frequency, the number of channels contributing to vesicle fusion only showed a small increase as vesicle fusion events continued to be driven by nanodomains (localized volume of high intracellular calcium ions near the mouth of a single open VGCC) of very few open VGCCs (Ma et al., 2015).

### The Impact of Active Zone Organization on Synaptic Function

**Figure 4:**
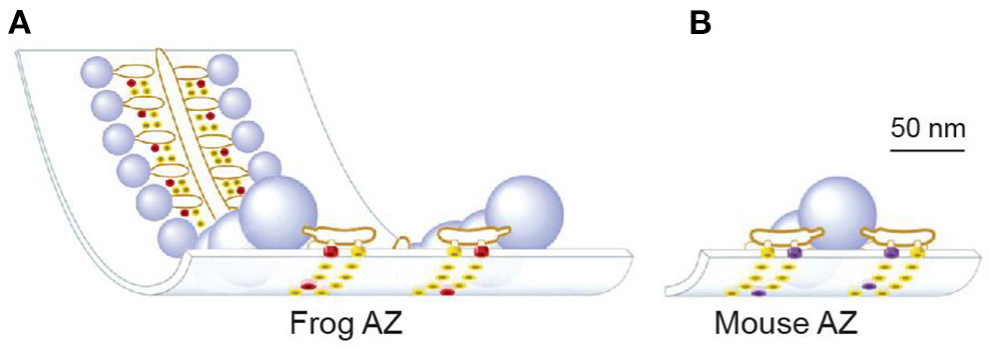
Drawings based on electron microscope tomography data of single active zone structure from frog **(A)** and mouse **(B)** neuromuscular junctions. Light blue spheres = docked synaptic vesicles; yellow dots represent AZ transmembrane proteins of unknown identity; red dots represent Cav 2.2 type VGCCs; purple dots represent Cav 2.1 type VGCCs. Adapted from Laghaei et al. (2018).

One important question that MCell modeling can address is the impact of differences in AZ structural organization on synaptic function. It is clear when one compares AZ structures from different synapses that there are many ways to organize these transmitter release structures (Zhai and Bellen, 2004). One of the most obvious aspects of AZ organization is the arrangement of VGCCs and docked synaptic vesicles, and it has been proposed that a docked synaptic vesicle and its associated VGCCs represent an essential unit building block of AZs (Tarr et al., 2013). The differential assembly of these building blocks into different complete AZ structures is hypothesized to lead to AZs with different functional properties (probability of release and short-term synaptic facilitation) (Tarr et al., 2013). Laghaei et al. (2018) tested this hypothesis by comparing the density and spatial organization of VGCCs and docked synaptic vesicles in frog vs. mouse neuromuscular AZs (Figure 4).

**Figure 5:**
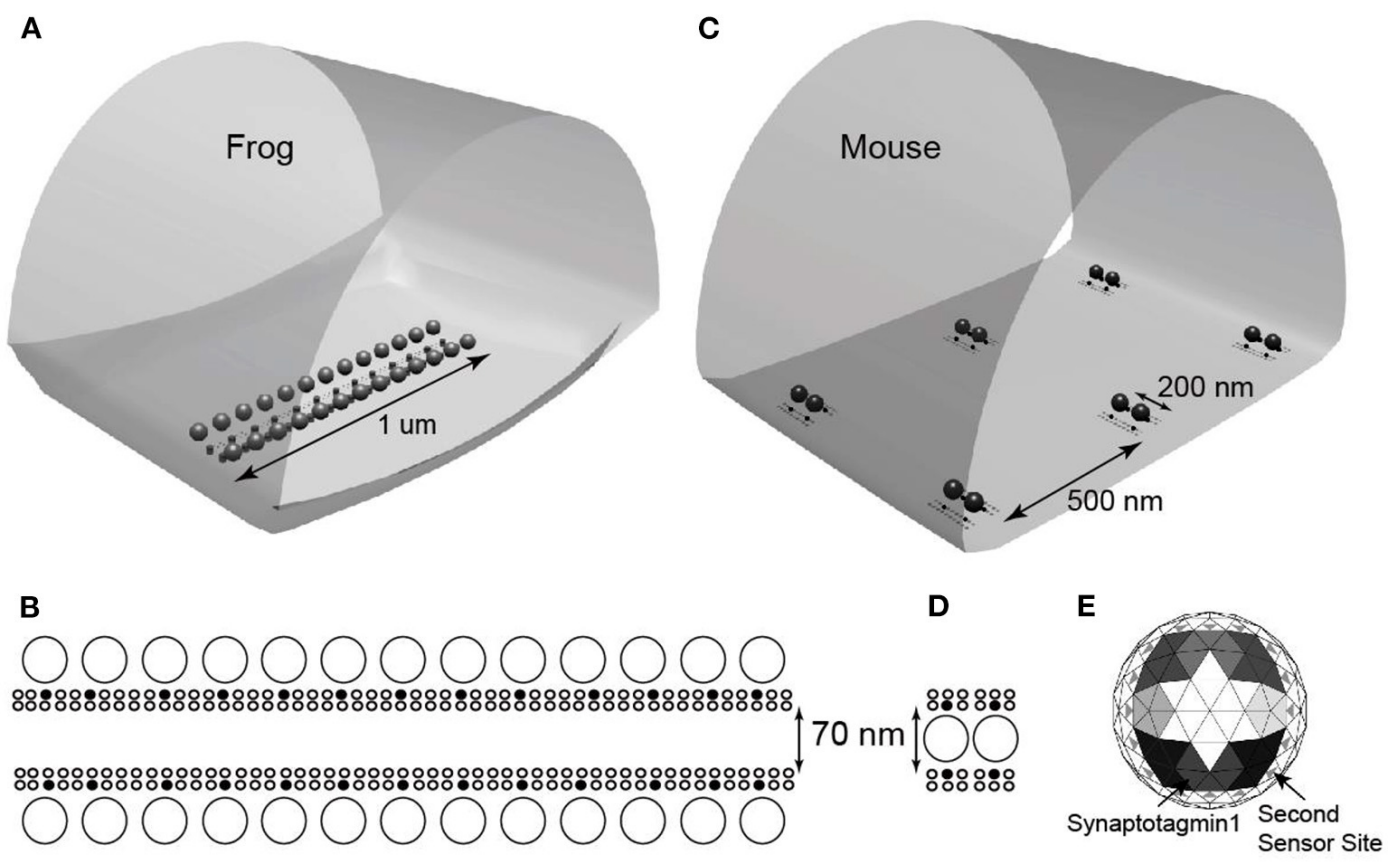
Representations of MCell models used for frog and mouse active zones. **(A)** The MCell geometry used to model the frog AZ. **(B)** Detail of AZ organization of docked synaptic vesicles (large open circles), VGCCs (small filled circles), and other AZ proteins (small open circles). **(C)** The MCell geometry used to model six mouse AZs. **(D)** Detail of AZ organization of docked synaptic vesicles (large open circles), VGCCs (small filled circles), and other AZ proteins (small open circles). **(E)** Diagram of the undersurface of docked synaptic vesicles. The model represents eight synaptotagmin-1 molecules (shaded large triangles; differences in shading depict the 5 binding sites for each of the 8 synaptotagmin-1 proteins), and 16 synaptotagmin-7 proteins (small gray triangles). Adapted from Laghaei et al. (2018).

Starting with a MCell model of the frog AZ that was validated by its ability to predict a large number of physiological phenomena at this synapse (probability of release, calcium-release relationship, temporal distribution of vesicle release events, and short-term synaptic plasticity), Laghaei et al. (2018) simply rearranged the VGCCs and docked synaptic vesicles from the frog pattern into the mouse pattern, without changing any other parameters in the model (Figure 5). This approach allowed them to isolate the role of AZ organization on synaptic function.

**Figure 6:**
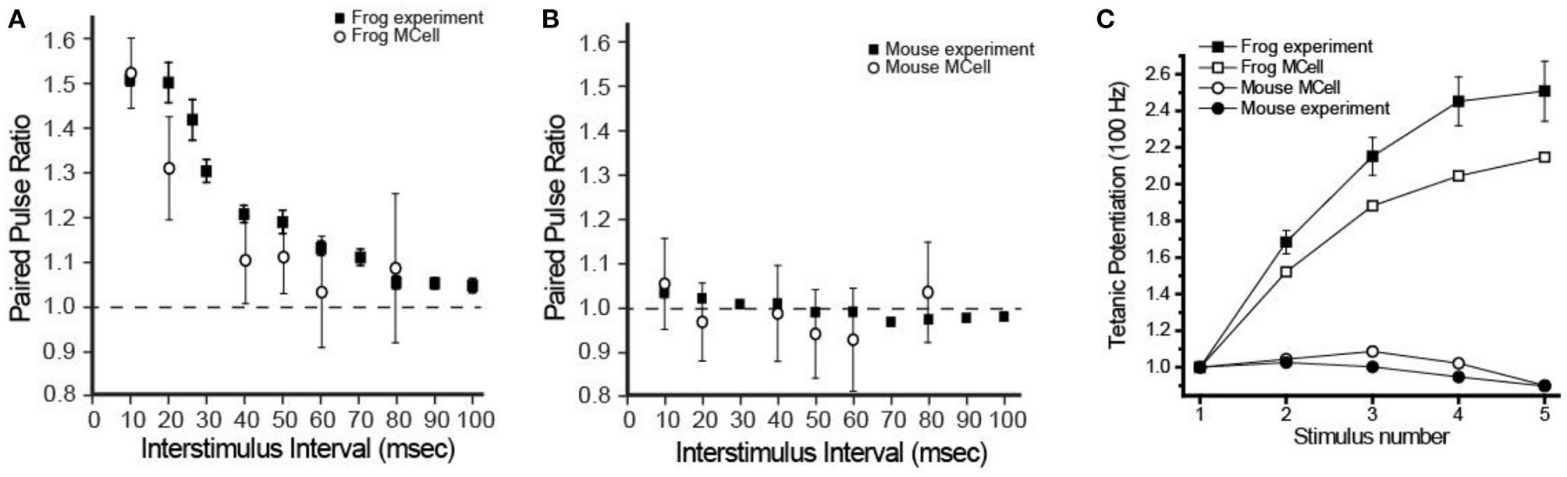
Comparison of physiological recordings and MCell model output of short-term synaptic plasticity in frog and mouse NMJs. **(A)** The paired-pulse facilitation (at different inter-stimulus intervals) observed at the frog NMJ can be reproduced the frog MCell model. **(B)** A simple rearrangement in the organization of elements in the frog MCell model into the organization observed in the mouse AZ predicts the lack of short-term plasticity during the paired-pulse protocol at different inter-stimulus intervals. **(C)** A plot of tetanic potentiation at 100 Hz in the frog (filled squares) and mouse (filled circles) is closely predicted by MCell simulation results of the frog AZ (open squares) and the mouse AZ (open circles) models. Adapted from Laghaei et al. (2018).

Functionally, the most striking physiological differences between a frog and mouse NMJ are the probability of release and short-term synaptic plasticity. Frog NMJs have a relatively low probability of release per docked synaptic vesicle (0.022), and very strong paired-pulse facilitation (1.51-fold change with a 10 ms interstimulus interval; ISI) and tetanic potentiation during 5 action potentials at 100 Hz stimulation (2.51-fold increase). Mouse NMJs have a stronger probability of release per docked synaptic vesicle (0.11), display no significant change during paired-pulse stimuli (1.03-fold with a 10 ms ISI), and mild tetanic depression during 5 action potentials at 100 Hz stimulation (10–20% reduction). Interestingly, using MCell models, all of these striking differences in function were predicted to emerge as a property of AZ organization (Figure 6) (Laghaei et al., 2018).

**Figure 7:**
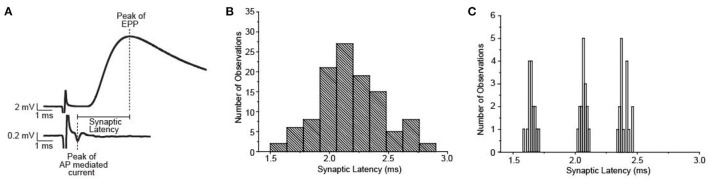
Synaptic delay at the frog NMJ is quite variable between synapses, but not within one synapse. **(A)** Representative data used to illustrate the method for calculating synaptic delay. **(B)** Plot of the average synaptic delays measured from 54 frog NMJs. **(C)** Plot of synaptic delay measured made from 3 of the 54 individual synapses shown in **(B)**. Repeated measurements (*n* = 16) made at each synapse demonstrate little variability within synapses but significant differences between synapses. Adapted from Homan et al. (2018).

Synaptic delay has been shown to be considerably variable across individual neuromuscular synapses at the frog NMJ (Figure 7), however, there are no experimental results that lend insight into the mechanisms that underlie this variability. Using a combination of intracellular electrophysiology experiments and MCell modeling, Homan et al. (2018) studied the impact of several AZ parameters on synaptic delay. These studies demonstrated which aspects of AZ organization and function could contribute to the variability in the synaptic delay across synapses at the frog NMJ.

The MCell modeling results from Homan et al. (2018) predict that synaptic delay is very sensitive to changes in extracellular calcium concentration, which highlights the importance of the intra-terminal spatiotemporal calcium profile. As might be expected based on this result, differences in buffer concentration can also have a strong impact on synaptic delay because calcium buffer is a key determinant of the intra-terminal spatiotemporal calcium profile. These MCell model results also predicted that differences in the distance between VGCCs and synaptic vesicles or removing significant numbers of VGCCs from the AZ had less of an impact on synaptic delay (Homan et al., 2018). The major conclusion from these studies is that intra-terminal calcium dynamics in the vicinity of docked synaptic vesicles is most likely to influence synaptic delay. This study highlights the ability of MCell modeling to explore different potential mechanisms that underly an experimental observation and provide insights into the sensitivity of each mechanism to impact the measured output. Using these modeling studies, investigators can design new experimental studies to explore potential mechanisms with greater insight into their impact.

## Author Contributions

RL and SM manuscript conceptualization and writing and editing of the manuscript. Both authors contributed to the article and approved the submitted version.

## Funding

This work was supported by NSF Collaborative Research in Computational Neuroscience Program Award Number: 2011616. This work used the Extreme Science and Engineering Discovery Environment (XSEDE), which was supported by National Science Foundation grant number ACI-1548562. Specifically, it used the Bridges-2 system, which was supported by NSF award number ACI-1928147, at the Pittsburgh Supercomputing Center (PSC).

## Conflict of Interest

The authors declare that the research was conducted in the absence of any commercial or financial relationships that could be construed as a potential conflict of interest.

## Publisher's Note

All claims expressed in this article are solely those of the authors and do not necessarily represent those of their affiliated organizations, or those of the publisher, the editors and the reviewers. Any product that may be evaluated in this article, or claim that may be made by its manufacturer, is not guaranteed or endorsed by the publisher.
